# The relations between symptoms, somatic and psychiatric conditions, life satisfaction and perceived health. A primary care based study

**DOI:** 10.1186/1477-7525-3-28

**Published:** 2005-04-27

**Authors:** Ahmad Al-Windi

**Affiliations:** 1 Family Medicine Stockholm, Karolinska Institute, Alfred Nobels allé 12, SE-141 83 Huddinge, Sweden

**Keywords:** depression, perceived health, Primary Care Evaluation of Mental Disorders (PRIME-MD), country of birth, health care practice, questionnaire.

## Abstract

**Background:**

In spite of the fact that self-rated health is such an important factor, little is known about the aetiological background to poor perceived health and also less is known about the impact of life satisfaction on health in a primary care practice population. The aim of this study was to evaluate the effect of socio-demographic characteristics, lifestyle factors, symptoms, somatic and psychiatric conditions as well as health status measures and life satisfaction on perceived health in a multi-ethnic Swedish health practice population.

**Methods:**

Four-hundred and seventy adult patients, who visited the Jordbro Health Care Centre District (JHC), Haninge Municipality, participated in this study. A general questionnaire with questions about socio-demographic characteristics, lifestyle, health status and chronic disease were used. In addition to that, the Primary Care Evaluation of Mental Disorders (PRIME-MD) was used. Furthermore, physical examinations were conducted. Unconditional logistic regression in successive models was used, adjusted for socio-demographic variables and other confounders.

**Results:**

Life satisfaction is the strongest predictor of poor perceived health in addition to country of birth, number of symptoms and depression. Being born in Sweden or other Nordic countries were related to lower OR as compared to those born outside Europe. The OR for non-depressed vs. depressed was 0.29 (0.17–0.48) and for non-symptomatic vs. symptomatic (1–3 symptoms) 0.25 (0.46–0.48). The OR and 95% CI for low satisfaction with life was 15.40 (5.28–44.97) in comparison to those who are satisfied with life.

**Conclusion:**

Country of birth, depression, number of symptoms and life satisfaction are factors related significantly and independently to perceived health. Life satisfaction is the strongest predictor of perceived poor health.

## Background

During the past two decades, interest in subjects' perceived health has become one of the important research fields in epidemiology and research concerning health services [1-4]. At the beginning of the 1980s, Kaplan and others reported an association between subjects' perceived health and mortality [5-7]. Several other international studies later confirmed these findings. Follow-up studies have shown similar results – i.e., poor self-rated health as a strong predictor of mortality [1,2,8]. Self-assessment of health status appears to be a good measure of current physical health, morbidity, disability and a predictor of health care utilisation [6,9-17].

Numerous reports have shown a strong association between various indicators of individual socio-economic positions [18-24]. A strong correlation has also been found between self-reported health status and both socio-economic status and increased risk of mortality for all ethnic groups [25]. In the United Kingdom a number of studies have found higher morbidity and mortality rates for most British ethnic minorities than in the white population [23,26-28]. A study from the United States observed that socio-economic status was a principal determinant of racial/ethnic disparities in health, but several other factors, for example, medical care, migration, stress and resources, also play a role [27]. However, the effect of ethnicity has been compared to social class [29].

It is evident that subjective health assessment is a valid indicator of health status in middle-aged populations, and that it can be used in cohort studies and monitoring of a population's health [3,25]. The use of socio-economic status has been recommended for screening of majority groups, but not for minority ones [30]. In spite of the fact that self-rated health is such an important factor, little is known about the aetiological background to poor perceived health and also less is known about the impact of life satisfaction on health in a primary care practice population. Palmore and Kivett reported in 1977 that life satisfaction at the end of a 4-year period was significantly related to initial levels of self-rated health among subjects aged 46–70 years [31]. However, some other studies on the elderly show relations between perceived health and life satisfaction [32-34]. For example, Wang et al. found that life satisfaction was related to mental health both in females and males [32]. Benyamini et al. reported from a study of an old population (mean age 73) that both self-rated oral health and self-rated health independently explained a significant amount in concurrent rates of self-esteem and life satisfaction [33]. Ho et al. found that satisfaction was related to higher social class, education, good perceived health and other factors [34].

The present investigation is part of a comprehensive programme entitled "Improving Health Care in Jordbro (IHCJ)" to assess the influence of socio-demographic characteristics, including country of birth, as well as morbidity on health care and drug utilisation in patients resident in Jordbro, a small multi-ethnic sub-community in Stockholm, Sweden. In this study we explore the relations between socio-demographic characteristics, lifestyle factors, somatic and psychiatric symptoms as well as health status measures and perceived health.

The aim of this study was to evaluate the effect of socio-demographic characteristics, lifestyle factors, symptoms, somatic and psychiatric conditions as well as health status measures, and life satisfaction on perceived health in a multi-ethnic Swedish health practice population. The Committee on Research Ethics at the School of Medicine, Karolinska Institute, approved the study.

## Methods

### Subjects and setting

A full description of the methodology is provided elsewhere [35]. The patient sample was recruited between October 2002 and April 2003 from adult patients (≥ 16 years old) presenting for routine visits at the Jordbro Health Care Centre (JHC). In total 470 adult patients who visited the JHC during the period participated in this study.

The JHC has a catchment area of 9 500 patients and approximately 9 000 patients are registered at the health centre. At the time of the study the proportion of patients >15 years old was 77%, i.e. 7 296 patients. The study population consists of patients who were registered consequently during the first four weeks of the study period and only six patients provided insufficient data, i. e. only six patients did not fulfil the survey completely. All adult patients (≥ 16 years) who visited the health care centre were included but not the emergency cases.

The study consisted of three parts: (a) a general questionnaire [35,36], (b) patient questionnaire (PQ) followed by a clinical interview [37] and (c) a medical examination.

a) The general questionnaire dealing with questions on socio-demographic characteristics, lifestyle, health status and medicine use was handed out to the patients. The socio-demographic variables included, in addition to age and gender, family situation (e.g. whether the patient lived alone, with another adult or with children) and country of birth. In addition to that, the questionnaire included questions on 16 common somatic diseases and one on whether the respondents perceived themselves to be healthy or not. They were also asked to indicate the degree of life satisfaction, perceived health and health status. Patients were asked to state whether they had any chronic disease.

b) The Primary Care Evaluation of Mental Disorders (PRIME-MD) questionnaire was used [37]. It is a two-stage process for diagnosing mental disorders by primary care physicians consisting of a patient questionnaire (PQ) as an initial screening followed by a structured clinical interview with a psychologist/physician, consisting of five diagnosing modules to further evaluate those groups of disorders whose presence had been indicated by positive answers to the initial questionnaire screening [37]. Psychiatric diagnoses were based on those listed in the *Diagnostic and Statistical Manual of Mental Disorders*, fourth edition (DSM-IV) [38]. A more detailed description of the PRIME-MD can be found in Loerch et al. [37].

c) Medical examination: this consisted of weight, height, and laboratory analyses including fasting blood glucose, serum cholesterol and serum triglycerides, flow volume spirometry and electrocardiography.

### Outcome variables

Subjects were asked to indicate their present degree of perceived health on a seven-point scale, ranging from score 1 "very bad" to score 7 "excellent, could not be better". The variables were dichotomised to "Good scores" score 5–7 and "Bad or poor scores (PPH)" score 1–4.

### Explanatory variables

#### The general questionnaire

The dichotomised or ordinal forms for these variables were used in nominal logistic regression – i.e., age was grouped into: 16–44, 45–64 and 65+. Gender: male and female. Working: yes and no. Country of birth: Sweden, other Nordic countries, Other European countries and rest of the world. *Living conditions: *the variables were: Living alone, Living with another adult and Living with children < 18 years. The presence of diagnosis was regarded as "yes", 1 and the "absence" as no, 0. Reported health status: *Healthy: *yes, 1; no, 0 and in between or unsure, 2. *Life satisfaction: *was defined on a seven-point scale, ranging from score 1 "very bad" to score 7 "excellent, could not be better". The variables were dichotomised to "Good scores" score 5–7, "Fair 'scores 3–4 and "Bad or poor scores" score 1–2.

### Psychiatric evaluation

*The patient questionnaire (PQ) *consisted of 28 yes/no questions about the presence of symptoms during the previous month, of which 15 were about somatic symptoms and the other about psychiatric symptoms and alcohol. *Psychiatric diagnoses*: mood disorders, anxiety, compulsive disorder, social phobia, probable alcohol abuse/dependence and eating disorders. The presence of diagnosis was regarded as "yes", 1, and the "absent" as no, 0.

### Medical Examination

BMI: Weight in kilograms/(height in metres)^2^; blood pressure: systolic and diastolic; heart rate (pulse) during 1 minute; fasting blood glucose, serum cholesterol and triglycerides. Spirometry: mean values were calculated for vital capacity (VC), forced expiratory volume in 1 second (FEV1), Forced vital capacity (FVC) and peak expiratory flow (PEF). Electrocardiography: The results were judged and grouped in four categories: normal, not surely pathological, suspect pathological and pathological. *Common somatic disease *consisted of 16 common conditions and one general question on chronic disease. The presence of condition was regarded as "yes", 1 and the absent as "no", 0.

### Statistical methods

The data were analysed with the SAS and JMP software packages [39,40]. Standard methods were used to obtain summary statistics, such as means, prevalence and other measures. Chi-square test or Fisher's exact test were used to calculate the p-values. Associations between perceived health and the continuous independent variables age, life satisfaction, BMI, blood pressure, heart rate, blood glucose, serum triglycerides and cholesterol were estimated as product moment correlations according to Spearman. All tests were two-tailed. Probability (p-) values less than 0.05 was regarded as statistically significant.

We analysed the relationships between perceived health and the explanatory variables, using the unconditional logistic regression in a successive model, adjusted for socio-demographic variables and other confounders. The interaction terms for chronic disease*depression, depression*symptoms and chronic disease*depression*symptoms were not significant.

The results are shown as odds ratios (OR) with 95% confidence intervals (CIs). The fit of the models was judged by the Hosmer-Lemeshow goodness-of-fit test. The models were considered acceptable if p > 0.05 and all models met this criteria. The results are shown as odds ratios (OR) with 95% confidence intervals (CIs).

## Results

### Socio-demographic characteristics, self-health report, health status measures, life-satisfaction and perceived health

Table 1 shows that 46.4% of respondents reported perceived poor health. All variables except living with or without children were significantly related to perceived health. A higher proportion of subjects aged 45–64 years reported poor health (PPH) as compared to younger subjects aged 16–44 years. The proportion of subjects reporting PPH is higher among females, subjects not working, subjects born outside Sweden, living alone and not living with another adult compared to males, working subjects, subjects born in Sweden, not living alone and living with another adult. A significantly higher proportion of subjects who report that they are not healthy or less satisfied with life have PPH in comparison with healthy and satisfied subjects. Life satisfaction was strongly correlated to perceived health (r = 0.66; p <.0001).

**Table 1:** Population distribution in per cent (%) by perceived health and sociodemographic characteristics, reported health status and life satisfaction.

		Perceived poor health (scores 1–4)		
		
Variable		N	%	P-value
Age				**<0.01**
1–44		120	40.8	
45–64		212	55.2	
65-		135	38.5	
Gender				**<0.001**
Men		169	36.1	
Women		298	52.8	
Working				**<0.005**
Yes		205	41.2	
No		252	52.8	
Country of birth				**<0.01**
Sweden		278	40.3	
Nordic		59	49.1	
Europe		58	67.2	
Other		72	52.8	
Living conditions				**<0.05**
Alone	Yes	128	57.8	
	No	336	42.6	
With other adult	Yes	318	41.5	**<0.005**
	No	146	58.2	
With children <18	Yes	123	42.3	NS
	No	341	48.1	
Healthy				**<0.0000**
Yes		247	25.1	
Fair		26	69.2	
No		190	72.1	
Life satisfaction				**<0.0001**
Low (score1–2)		45	88.9	
Fair (score 3–4)		128	78.9	
High (score 5–7)		292	25.7	
Population		470	46.4	

* N not equal to 470 due to missing some values.

Apart from BMI and smoking status, no other variable was significantly related to perceived health, table 2. Subjects who report PPH have higher BMI and smoke more than those with perceived good health. BMI is negatively related to PPH. The correlation for BMI and PPH is (r = -0.10; p < .05).

**Table 2:** Population distribution in per cent (%) by perceived health and health status measures. Values are means unless otherwise indicated. Poor perceived health (N = 218), good (N = 250).

		Perceived health			
		
Variables		Number	Poor (scores 1–4)	Good (scores 5–7)	P-value
Body Mass Index (BMI)*			28.3	27.3	**<0.05**
Smoking	Yes	133	57.1	42.9	**<0.005**
	No	335	42.3	57.7	
Blood pressure					
Systolic BP			134.4	135.4	NS
Diastolic BP			80.5	80.0	NS
Heart rate/minute			70.7	68.8	NS
Blood glucose			5.99	5.78	NS
Serum triglycyrids			1.54	1.50	NS
Serum cholesterol			5.46	5.28	NS
Spirometry					
Vital capacity (VC)			96.5	93.8	NS
Forced expiratory volume (FEV1)			92.6	93.9	NS
Forced vital capacity (FVC)			91.8	91.3	NS
PEF			89.3	91.8	NS
Electrocardiography**					NS
Normal		72	52.8	46.2	
Not sure pathological		49	36.7	63.3	
Suspect pathologic		226	43.8	56.2	
Pathological		111	52.3	47.8	

* BMI = Weigh/length (m^2^)

**Per cent

### Symptoms and perceived health

Having any of the 13 of 15 symptoms listed in the PQ was significantly related to PPH, i.e. those who report any of these symptoms more often have PPH than do non-symptomatic subjects. For some of these symptoms the risk is almost doubled or tripled, Table 3. For example, 87% of those reporting fainting spells have PPH compared to 44.4% among non-symptomatic subjects. The figures for feeling tired or having low energy and trouble sleeping were 62.6 and 62.9% as compared to 26.2 and 33.6% respectively.

**Table 3:** The number of subjects with/without symptoms and the percentage (%) of these reporting perceived poor health (PPH).

	Number*		PPH %		P-value
Symptoms	Yes	No	Yes	No	
Stomach pain	130	339	60.3	41.3	**<0.0005**
Back pain	218	251	59.0	35.5	**<0.0001**
Pain in arms, legs & joints	288	181	52.4	37.0	**<0.005**
Menstrual cramps	46	210	50.0	46.2	NS
Headaches	176	291	57.0	40.2	**<0.001**
Chest pain	100	368	66.7	41.0	**<0.0001**
Dizziness	133	333	64.9	39.3	**<0.0001**
Fainting spells	23	446	87.0	44.4	**<0.0001**
Palpitations	88	381	63.6	42.5	**<0.001**
Shortness of breath	86	383	65.1	42.3	**<0.0005**
Pain or problems during sexual intercourse	27	238	57.7	45.4	NS
Constipation, loose bowels, or diarrhoea	117	352	62.4	41.2	**<0.0001**
Nausea, gas, or indigestion	145	324	58.6	41.1	**<0.001**
Feeling tired or having low energy	263	206	62.6	26.2	**<0.0001**
Trouble sleeping	202	265	62.9	33.6	**<0.0001**

* N not equal to 470 due to missing some values.

### Chronic disease or conditions and perceived health

Subjects who report having any chronic disease or condition report to a higher extent having PPH, table 4. In general about 50% of subjects with chronic disease report PPH as compared to 26.3% among non-diseased subjects. Having any of nine of these diseases or conditions was related to PPH, heart failure, asthma, neurological disease, musculoskeletal and joint disorders, pain syndrome, psychiatric disorders, gastrointestinal and urinary tract troubles. For example, about 80% of subjects with a psychiatric diagnosis report PPH and for heart failure the figure is 73.3%.

**Table 4:** The number of subjects with/without a disease or a condition and the percentage (%) of these reporting perceived poor health (PPH).

	Number*		PPH %		
Disease or condition	Yes	No	Yes	No	P-value
Chronic disease	394	76	50.3	28.5	**<0.005**
Blood pressure	84	354	49.6	45.8	NS
Angina	38	429	52.6	46.1	NS
Heart failure	15	452	73.3	45.8	**<0.01**
Diabetes	44	422	54.5	45.7	NS
Asthma	59	408	67.8	43.6	**<0.005**
Chronic obstructive disease	13	454	38.5	46.9	NS
Neurological disease	25	441	68.0	45.6	**<0.0005**
Musculoskeletal disease	89	378	68.5	41.5	**<0.0001**
Joint disease	255	212	55.3	36.3	**<0.0005**
Pain syndrome	112	355	65.1	39.4	**<0.0001**
Cancer	18	448	44.4	46.9	NS
Psychiatric disorder	40	427	80.0	43.6	**<0.0001**
Eye disease	65	402	53.8	45.5	NS
Ear disease	39	427	46.1	46.8	NS
Gastrointestinal disorders	120	347	59.2	42.4	**<0.0005**
Urinary tract disease	59	406	55.9	45.1	**<0.05**

* N not equal to 470 due to missing some values.

From table 5 is obvious that depression, anxiety and compulsive disorders are related to PPH. About 75% of subjects with one of these three disorders report PPH.

**Table 5:** The number of subjects with/without a psychiatric diagnoses and the percentage (%) of these reporting perceived poor health (PPH). Perceived poor health (N = 218), good (N = 250).

	Number		% PPH		
Diagnose	Yes	No	Yes	No	P-value
Depressive disorders	216	249	73.8	34.6	**<0.0001**
Anxiety	216	380	76.5	39.7	**<0.0001**
Compulsive disorders	23	441	76.5	44.4	**0.0001**
Social phobia	10	455	60.0	46.2	NS
Probable alcohol abuse/dependent	32	433	46.9	46.4	NS
Eating disorders	11	454	54.5	46.3	NS

### Logistic regression analyses

Table 6 shows odds ratios (OR) with 95% confidence intervals (95% CI) for having poor health by age, gender, living and working status, country of birth, smoking, chronic disease, depression, number of symptoms and life satisfaction. The ORs are adjusted for all confounders. We analysed the relationships between perceived health and the explanatory variables, using the unconditional logistic regression in successive models, adjusted for age and gender in the first model. This shows that subjects aged 45–64 years have higher OR than older subjects, i.e. aged 65 or older. The OR was 1.62. The figure for males was 0.52 compared to females.

**Table 6:** Odds rations (OR) with 95% confidence intervals (95% CI) for having poor health by age, gender, living and working status, country of birth, smoking, chronic disease, depression, number of symptoms and life satisfaction. The ORs are adjusted for all confounders.

		Perceived poor health (scores 1–4)							
		**Model 1**		**Model 2**		**Model 3**		**Model 4**	
		OR	95% CI	OR	95% CI	OR	95% CI	OR	95% CI
Age (65+ years = reference)	16–44	0.96	0.59–1.57	1.51	0.83–2.76	0.65	0.31–1.33	0.65	0.29–1.45
	45–64	**1.62**	**1.05–2.50**	**2.47**	**1.45–4.21**	1.73	0.95–3.18	1.44	0.74–2.81
									
Male (female= ref)		0.52	0.35–0.76	0.56	0,37–0.84	0.86	0.54–1.37	0.87	0.52–1.46
									
Living alone (no = ref)				**0.52**	**0.33–0.83**	0.67	0.40–1.11	0.74	0.42–1.30
Working (yes =ref)				**2.10**	**1.35–3.26**	1.42	0.86–2.34	1.21	0.69–2.11
Country of birth (other = ref)	Sweden			**0.50**	**0.28–0.88**	0.52	0.26–1.02	**0.41**	**0.20–0.84**
	Nordic			0.57	0.27–1.21	0.44	0.19–1.02	**0.26**	**0.10–0.66**
	Europe			1.19	0.55–2.57	0.96	0.40–2.31	0.68	0.26–1.80
									
Smoking (yes = ref)						0.81	0.50–1.32	0.97	0.56–1.66
Chronic disease (yes = ref)						0.68	0.36–1.27	0.97	0.56–1.27
Depression (yes = ref)						**0.29**	**0.17–0.48**	**0.55**	0.31–0.98
Number of symptoms (>6 ref)	1–3 symptoms					**0.25**	**0.13–0.48**	**0.30**	0.15–0.60
	4–6 symptoms					0.85	0.46–1.58	0.98	0.50–1.92
									
Life satisfaction* (high = ref)	Low							**15.40**	**5.28–44.97**
	Fair							**7.02**	**3.98–12.38**

*Low (scores 1–2), fair (scores 3–4) and high (scores 5–7)

** Interaction tests for chronic disease*depression, depression*symptoms and chronic disease*depression*symptoms were not significant.

In the second model, when living conditions and country of birth were added to the analysis, the OR for subjects aged 45–64 years and for males remained significant. The ORs for subjects not living alone, working subjects and those born in Sweden were lower than for those living alone, not working and born outside Europe.

In the third model, with the addition of smoking status, chronic disease, depression and number of symptoms, the pattern changed. Neither age nor gender was significant any longer. However, depression and number of symptom were significant. Non-depressed had OR = 0.29 as compared to depressed. This means that depressed people have 71% higher risk than non-depressed of having PPH. The OR for having 1–3 symptoms and PPH was 0.25 as compared to those with more than 6 symptoms. The OR for those with 4–6 symptoms was 0.85, as compared to those with more than 6 symptoms, but this is not significant. The linear trend is interesting. However, the OR for subjects born Sweden was still lower as than for those born outside Europe but the 95% CI overlapped 1 (1.02), which was not significant.

In the final model (model 4) when all confounders were included, i.e. life satisfaction in addition to the previous variables, being born in Sweden or other Nordic countries was related to lower OR as compared to those born outside Europe. The OR for those born in Sweden was 0.41 and for those born in other Nordic countries 0.26. The OR for non-depressed and those with 1–3 symptoms was somewhat higher but still significant.

The OR for low satisfaction with life was as high as 15.40 in comparison to those who are satisfied with life. However, the 95% CI was very wide, 5.28–44.97. The OR for those who report fair life satisfaction is 7.02 and the 95%CI is 3.98–13.38.

## Discussion

This study has shown that various socio-demographic characteristics and country of birth affect the subject's perceived health. A substantially high percentage reported that they had poor perceived health, with the lowest percentage in those born in Sweden. These figures were higher than the national Swedish average (5–6%) and in other international reports [41,42]. However, Hjern et al. also found a higher prevalence of poor perceived health in subjects born outside Sweden [43]. Williams, in a study of women from the United States, found that socio-economic status was an important determinant of racial/ethnic disparities in health, but several other factors including, for example, medical care, migration, acculturation, stress and resources, also play a role [27]. Not surprisingly, perceived health was influenced by socio-demographic characteristics in this study, but it is noteworthy that another individual factor, "life satisfaction", showed a profile stronger than that of socio-demographic characteristics even after adjustment was made for age, gender, education, occupation, country of birth, chronic disease or condition and symptoms. This factor probably plays an important role in a subject's health and indicates the overall life satisfaction including health. The question about "life satisfaction" can also be used to assess health. Prospective studies are needed to confirm this. Indeed, this variable can probably be used together with perceived health, but needs to be investigated further. Many studies have shown the effect of age, gender, education, occupation and country of birth on health, which accord with our results [44,45,5].

An interesting finding in this study is that patients aged 45–64 years have poorer health than older subjects. We believe that in this multi-ethnic population this age group is more vulnerable and sicker than the older subjects. It is possible that specific factors related to the neighbourhood in this study have an impact on health. Future studies should elucidate this issue. This could have important health policy implications. However, in the logistic model, the impact of age was not any longer significant when the symptom variable was taken into account.

As is the case with all questionnaire surveys, there was the possibility that patients exaggerated or underreported a condition, partly due to difficulties in remembering (recall bias). In addition to the potential problem of recall bias, there is the possibility of selection bias. The participants in this investigation were voluntary and time-limited, and we could only include those who showed interest first. It is possible that some selection bias occurred as a result of this consecutive procedure. It is possible, for instance, that our study included only the healthier patients in the Jordbro Health District because the sickest patients were not able to come to the health centre.

The questionnaire used in the present study (PRIME-MD) has been validated previously and shown to have good accuracy for psychiatric disorders [51-53]. Also, different parts of the questionnaire used here were previously validated in other investigation [35,36,49,51]. We also tested the questionnaire as a whole in advance in a pilot study and judged it to be satisfactory.

Although this study is not prospective, causal relations cannot be drawn from this investigation. The most of the literature on this issue are cross-sectional studies and not suited for statement or implications. Further prospective research is needed to clarify the direction of association. The new finding in this study is the strong association between life satisfaction and perceived health apart from country of birth, symptoms and depression, and also the fact that perceived health has a stronger correlation with psychiatric than somatic conditions. The strength of this study is that this represents a primary care patient population which makes it unique. Furthermore, the fact that we have controlled for a large number of confounders makes it more unique.

In conclusion, country of birth, depression, number of symptoms and life satisfaction are factors related significantly and independently to perceived health. Life satisfaction was the strongest predictor of poor health.

## Abbreviations

OR = Odds ratio

95% CI = 95% Confidence Interval

Prime-MD = Primary Care Evaluation of Mental Disorders

PQ = Patient questionnaire

JHC = Jordbro Health Care Centre

VC = Vital capacity

FEV1 = Forced vital capacity during one second

PEF = Peak expiratory flow

PPH = poor perceived health

r = Spearman's correlation coefficient

BMI = body mass index

## References

[B1] Kaplan GA, Camacho T (1983). Perceived health and mortality: a nine-year follow-up of the Human Population Laboratory cohort. Am J Epidemiol.

[B2] Sundquist J, Johansson SE (1997). Self-reported poor health and low educational level predictors for mortality: a population-based follow-up study of 39,156 people in Sweden. J Epidemiol Community Health.

[B3] Miilunpalo S, Vuori I, Oja P, Pasanen M, Urponen H (1997). Self-rated health status as a health measure: the predictive value of self-reported health status on the use of physician services and on mortality in the working-age population. J Clin Epidemiol.

[B4] Idler EL, Angel RJ (1990). Self-rated health and mortality in the NHANES-1 epidemiologic follow-up study. Am J Public Health.

[B5] Kaplan G, Barell V, Lusky A (1988). Subjective state of health and survival in elderly adults. J Gerontol.

[B6] Idler EL, Benyamini Y (1997). Self-rated health and mortality: a review of twenty-seven community studies. J Health Soc Behav.

[B7] Benyamini Y, Idler EL (1999). Community studies reporting associations between self-rated health and mortality. Research in Aging.

[B8] Heistaro S, Jousilahti P, Lahelma E, Vartiainen E, Puska P (2001). Self-rated health and mortality: a long-term prospective study in eastern Finland. J Epidemiol Community Health.

[B9] Angel R, Gronfein W (1988). The use of subjective information in statistical models. ASR.

[B10] Wannamethee G, Shaper AG (1991). Self-assessment of health status and mortality in middle-aged British men. Int J Epidemiol.

[B11] Ferraro KF, Farmer MM, Wybraniec JA (1997). Health trajectories: Long-term dynamics among black and white adults. J Health and Soc Behav.

[B12] Al-Windi A, Elmfeldt D, Svärdsudd K (2004). The influence of sociodemographic characteristics on health care utilisation in a Swedish municipality. Ups J Med Sci.

[B13] Idler EL, Kasl S (1995). Self-ratings of health: Do they also predict change in functional ability?. J Gerontol B Psychol Sci Soc Sci.

[B14] Al-Windi A, Elmfeldt D, Svärdsudd K (2002). The influence of perceived well-being and reported symptoms on health care utilization: A population-based study. J Clinical Epidemiol.

[B15] Connelly JE, Smith GR, Philbrick JT, Kaiser DL (1991). Healthy patients who perceive poor health and their use of primary care services. J Gen Intern Med.

[B16] Kjoller M, Rasmussen NK, Keiding LM (1999). Self-reported health and morbidity among adult Danes 1987–1994. Ugeskr Laeger.

[B17] Borgquist L, Hansson L, Nettelbladt P, Nordstrom G, Lindelow G (1993). Perceived health and high consumers of care. a study of mental health problems in a Swedish primary health care district. Psychol Med.

[B18] Sihvonen AP, Kunst AE, Lahelma E, Valkonen T, Mackenbach JP (1998). Socioeconomic inequalities in health expectancy in Finland and Norway in the late 1980s. Soc Sci Med.

[B19] Hallqvist J, Lundberg M, Diderichsen F, Ahlbom A (1998). Socioeconomic differences in risk of myocardial infarction 1971–1994 in Sweden: Time trends, relative risks and population-attributable risk. Int J Epidemiol.

[B20] Cavelaars AE, Kunst AE, Geurts JJ, Helmert U, Lundberg O, Mielck A, Matheson J, Mizrahi A, Mizrahi A, Rasmussen N, Spuhler T, Mackenbach JP (1998). Morbidity differences by occupational class among men in seven European countries: an application of the Erikson-Goldthorpe social class scheme. Int J Epidemiol.

[B21] Devlin N, Hansen P, Herbinson P (2000). Variations in self-reported health status: results from a New Zealand survey. N Z Med J.

[B22] Morris JK, Cook DG, Walker M, Shaper AG (1992). Non-consulters and high consulters in general practice: cardio-respiratory health and risk factors. J Public Health Med.

[B23] Marmot MG, Adelstein AM, Bulusu L (1984). Immigrant mortality in England and Wales 1970–78. Causes of death by country of birth. Studies on Medical and Population Subjects No. 47.

[B24] Nazroo JY, Bartley MB, Blane D, Davey Smith G (1998). Genetic, cultural or socio-economic vulnerability? Explaining ethnic inequalities in health. The sociology of health inequalities.

[B25] McGee DL, Liao Y, Cao G, Cooper RS (1999). Self-reported health and mortality in a multiethnic US cohort. Am J Epidemiol.

[B26] Harding S, Maxwell R (1997). Differences in the mortality of migrants. In F. Drever, M Whitehead, Health inequalities.

[B27] Williams DR (2002). Racial/ethnic variations in women's health: the social embeddedness of health. Am J Public Health.

[B28] Nazroo JY (1997). The health of Britain's ethnic minorities.

[B29] Sundquist J (1995). Ethnicity, social class and health. A population-based study on the influence of social factors on self-reported illness in 223 Latin American refugees, 333 Finnish and 126 southern European labour migrants and 841 Swedish controls. Soc Sci Med.

[B30] Kwok RK, Yankaskas BC (2001). The use of census data for determining race and education as SES Indicators: A validation study. AEP.

[B31] Palmore E, Kivett V (1977). Change in life satisfaction: a longitudinal study of persons aged 46–70. J Gerontol.

[B32] Wang CW, Iwaya T, Kumano H, Suzukamo Y, Tobimatsu Y, Fukudo S (2002). Relationship of health status and social support to the life satisfaction of older adults. Tohoku J Exp Med.

[B33] Benyamini Y, Leventhal H, Leventhal EA (2004). Self-rated oral health as an independent predictor of self-rated general health, self-esteem and life satisfaction. Soc Sci Med.

[B34] Ho SC, Woo J, Lau J, Chan SG, Yuen YK, Chan YK, Chi I (1995). Life satisfaction and associated factors in older Hong Kong Chinese. J Am Geriatr Soc.

[B35] Al-Windi A Depression in general practice. Nord J Psychiatry.

[B36] Tibblin G, Peciva S, Kullman S, Svardsudd K (1990). "The Gothenburg quality of life instrument": An assessment of well-being and symptoms among men born 1913 and 1923. Methods and validity. Scand J Prim Health Care.

[B37] Loerch B, Szegedi A, Kohnen R, Benkert O (2000). The Primary Care Evaluation of Mental Disorders (PRIME-DM) German version: a comparison with CIDI. J Psychiat Res.

[B38] (1994). American Psychiatric Association. Diagnostic and statistical manual of mental disorders.

[B39] (1997). SAS: Introductory Guide; ed 3.1. Campus Drive, SAS Institute 31 ed Cary.

[B40] (2000). JMP-program:. Statistical Discovery Software Introductory Guide, Version 4 SAS Institute Inc, Cary, NC, USA.

[B41] Statistics (2002). Livslängd, hälsa och sysselsättning (Length of life, health and occupation). Stockholm, Sweden, Statististka centralbyrån (Statistics Sweden).

[B42] Markovic M, Manderson L, Kelaher M (2002). The health of immigrant women: Queensland women from the former Yugoslavia. J Immigrant Health.

[B43] Hjern A, Haglund B, Persson G, Rosen M (2001). Is there equity in access to health services for ethnic minorities in Sweden?. Eur J Public Health.

[B44] Eriksson I, Undén A-L, Elofsson S (2001). Self-rated health. Comparisons between three different measures. Results from a population study. Int J Epidemiol.

[B45] Verbrugge LM (1982). Sex differences in health. Prevention.

[B46] Gijsbers van Wijk CM, Kolk AM (1997). Sex differences in perceived health. Ned Tijdschr Geneeskd.

[B47] Joung IM, van der Meer JB, Mackenbach JP (1995). Marital status and health care utilization. Int J Epidemiol.

[B48] Fylkenes K (1993). Determinants of health care utilisation: Visits and referrals. Scand J Soc Med.

[B49] Al-Windi A (2000). Determinants of Health Care and Drug Utilisation: The Causes of Health Care Utilisation Study. Uppsala, Uppsala University, PhD Thesis.

[B50] Bosworth HB, Butterfield MI, Stechuchak KM, Bastian LA (2000). The relationship between self-rated health care service use among women veterans in a primary care clinic. Women's Health Issues.

[B51] Parker T, May PA, Maviglia MA, Petrakis S, Sunde S, Gloyd SV (1997). PRIME-MD: its utility in detecting mental disorders in American Indians. Intl J Psychiat Med.

[B52] Kobak KA, Taylor LH, Dottl SL, Greist JH, Jefferson JW, Burroughs D, Mantle JM, Katzelnick DJ, Norton R, Henk HJ, Serlin RC (1997). A computer-administered telephone interview to identify mental disorders. JAMA.

